# Using latent profile analysis to understand palliative care professionals’ quality of life during the COVID-19 pandemic

**DOI:** 10.1007/s12144-022-03958-3

**Published:** 2022-11-11

**Authors:** Cristina Lluch-Sanz, Laura Galiana, José M. Tomás, Amparo Oliver, Gabriel Vidal-Blanco, Noemí Sansó

**Affiliations:** 1 Department of Methodology for the Behavioral Sciences, University of Valencia, Av. Blasco Ibañez, 21, 46010 Valencia, Spain; 2 Department of Nursing, University of Valencia, Valencia, Spain; 3 Department of Nursing and Physiotherapy, University of the Balearic Islands, Cra. de Valldemossa, Km 7.5, 07120 Palma, Spain; 4 Balearic Islands Health Research Institute (IDISBA), Palma, Spain

**Keywords:** Burnout, Work satisfaction, Compassion fatigue, Compassion satisfaction, Healthcare personnel, COVID-19

## Abstract

Healthcare workers’ professional quality of life has been increasingly under the spotlight, even more so during the COVID-19 pandemic, which has posed a genuine challenge for them. This study aims to describe the professional quality of life profiles of a sample of Spanish palliative care professionals during the COVID-19 pandemic, encompassing aspects such as work satisfaction, burnout, compassion fatigue, and compassion satisfaction; while studying the relationships between these profiles and sociodemographic variables, clinical situations experienced during the pandemic, protectors of professional quality of life, the quality of care delivered, and the professionals’ wellbeing. Data from a survey of Spanish palliative care professionals were used. The variables measured were professional quality of life, sociodemographic characteristics, COVID-19-related experiences, protectors of professional quality of life, wellbeing, and quality of care. Our research included latent profile analyses, along with chi-squared and *t*-tests. The results suggested two profiles of professional quality of life, namely low (32.78%) and high (67.22%). The following profile displayed a higher likelihood of having a low professional quality of life: younger professionals, registered nurses, with a decrease in their teamwork, without specific training in palliative care, in coping with death and stress or emotional training and with lower levels of self-care and self-compassion, whose patients were unable to die a dignified death. Similarly, a low professional quality of life profile was associated with reduced wellbeing and poorer quality of care offered. In conclusion, providing professionals with education and training to improve their ability to handle end-of-life care and stress, maintaining cohesive teams and promoting self-care and self-compassion are pivotal to maintaining the quality of life and wellbeing of palliative care professionals and the quality of care that they provide.

## Background

### Healthcare workers’ professional quality of life

Healthcare workers’ professional quality of life of in general, and more notably those providing palliative care professionals, is a topic of growing interest in both the scientific and clinical fields. The traditional literature on professional quality of life has identified two main aspects of this phenomenon: work satisfaction, the positive side, and burnout, the negative side. Work satisfaction has been defined as a positive emotional state experienced by a worker about his or her job or work experience (Locke, 1976). This emotional state has been linked to the fulfilment of the worker’s needs, and includes a subjective assessment derived from two main comparisons: one between worker expectations vis-à-vis the challenges they face, and the other between the rewards the worker receives versus what they feel they should receive (Goh et al., 2015). Burnout, on the other hand, is a syndrome appearing in stressful situations that is experienced primarily by human service employees (Freudenberger, 1974). Healthcare professionals are particularly prone to this since their working environment is characterised by high-risk decision-making, dealing with the public, and expectations of compassion and sensitivity (Burton et al., 2017).

However, studies have shown that work satisfaction and burnout alone do not explain professionals’ emotional issues arising from working with traumatised individuals (Figley, 1995). It is against this backdrop that compassion fatigue has increasingly come to the fore in recent years. Compassion fatigue may be defined as the negative effects of working with traumatised individuals (Stamm, 2010), and focuses specifically on the chronic concern and stress produced by continued exposure to traumatised individuals (Figley, 1995). The term compassion fatigue characterises a state of diminished capacity for compassion as a consequence of being exhausted due to dealing with the suffering of others (Figley, 2002). Within this framework, evidence suggests that compassion fatigue can lead to the development of psychological difficulties, physical and emotional exhaustion, an inability to provide compassion, and a reduced capacity to withstand the suffering of others (Figley, 1995, 2002).

Research on compassion fatigue has also defined its opposite or inverse effect, i.e. compassion satisfaction. Compassion satisfaction takes place when exposure to traumatic events produces a sense of gratification due to the joy of helping others and provides a means to alleviate suffering that results in feelings of satisfaction (Hooper et al., 2010). Indeed, when helping individuals and changing their lives is properly managed, professionals and caregivers can experience pleasure and satisfaction rather than compassion fatigue and burnout (Figley & Stamm, 1996). These dimensions are key in the therapeutic relationship in the presence of suffering.

As some authors state, the concepts of compassion fatigue and compassion satisfaction are currently one of the dominant theoretical frameworks in studies, which examine the consequences of caring for others (Geoffrion et al., 2019). In this context, the professional quality of life (ProQOL) framework as proposed by Stamm (2010) is one of the most (if not the most) used. This model aims to explain the consequences of working as a “helper” to traumatized individuals, consequences many time negative, such as the aforementioned burnout and compassion fatigue, but also positive ones, such as compassion satisfaction. Taking into account Stamm’s contributions (2010), and also more traditional aspects of professional quality of life, such as work satisfaction, in this study we will conceptualize the professional quality of life of healthcare professionals as the interrelation of four constructs, two of them in negative terms, burnout and compassion fatigue, and two in positive terms, work satisfaction and compassion satisfaction.

### Palliative care workers’ professional quality of life during COVID-19 pandemic

Faced with this scenario, the COVID-19 pandemic has entailed a real challenge to professionals’ quality of life, both in terms of its traditional conceptualisation and in the one outlined in the foregoing section. Healthcare professionals have been exposed to the front line of the pandemic, which has also meant that they have suffered the most damaging consequences. Several authors have highlighted potentially harmful factors for healthcare professionals, including the lack of access to adequate protective equipment (Pfefferbaum & North, 2020); the exhaustion of wearing personal protective equipment for the entire working shifts or feeling inadequately supported (Liu et al., 2020); prolonged working hours and unexpected changes in the type of work demanded of them (Galli et al., 2020); the lack of access to updated information on constantly changing guidelines for action (Liu et al., 2020); or the uncertainty regarding disease containment (Pfefferbaum & North, 2020).

Alongside this set of factors that have affected all healthcare professionals, palliative care professionals have faced two further additional threats, or lacked two working conditions that have been essential to them since almost the dawn of palliative care: changes to teamwork and peer support, and patients who die without family support or adequate end-of-life care. The main goal of palliative care professionals is to meet patients’ needs, including not solely on a physical level, but also their psychological, social, and spiritual needs. To achieve this, professionals build a therapeutic relationship with patients and rely on teamwork, two of the main values of this care model. The health crisis emanating from the current COVID-19 pandemic has often reduced the capacity of professionals to attend to many of the emotional and spiritual needs of patients by not being able, for example, to allow them to die surrounded by their loved ones. The pandemic has also hindered appropriate symptom management, discussions with patients about their wishes, and the provision of emotional and spiritual support. For instance, Mitchinson et al. (2021) found evidence showing that palliative care professionals struggled to connect with patients due to increased work pressures and limited opportunities for human interaction.

Adding to this issue is the fact that, in most services, the dynamics of work teams has been shifting due to changes in their composition, as well as changes to tasks and protocols. Indeed, work reorganization has emerged as an important theme when studying the impact of the COVID-19 pandemic in palliative care workers (Pastrana et al., 2021). This reorganization of work has involved the restructuring of work teams, a cornerstone in the context of palliative care. In the healthcare context, teamwork has been repeatedly associated to improved care quality and safety, patient satisfaction, health outcomes, increased job commitment and work engagement, decreased employee injury, intent to leave, and, specifically, to decreased burnout (Rosen et al., 2018). Specifically in the palliative care literature, a recent work on this topic has pointed how collaborative teamwork, both within and between specialized palliative care services and with other generalist palliative care providers, has arisen as an enabler to implement changes and innovations in response to the health crisis (Dunleavy et al., 2021).

In sum, and in addition to the well-known circumstances that have affected healthcare professionals during the COVID-19 crisis, palliative care professionals have faced two circumstances: patients who die without family support or adequate end-of-life care, and changes to teamwork and peer support. These circumstances may lead to a negative bearing on professional performance (Fernando & Hughes, 2019), though similarly on professional quality of life (Moreno-Mulet et al., 2021).

### Predictors of palliative care workers’ professional quality of life

Other variables that have been linked to the professional quality of life of healthcare workers during the pandemic include gender, age and discipline. For example, women have reported higher rates of burnout compared to men in several studies conducted during the pandemic (Duarte et al., 2020). In the same vein, studies into healthcare professionals reported higher levels of compassion fatigue among women (Samaniego et al., 2020). In terms of disciplines, nurses reported higher scores on burnout scales in several studies (Chor et al., 2021). However, other authors have reported higher levels of burnout among clinicians (Dosil et al., 2020), along with higher levels of compassion fatigue and lower levels of compassion satisfaction (Ruiz-Fernández et al., 2020).

Finally, a number of factors have traditionally been linked to the professional quality of life of palliative care professionals, although deep-rooted study into their role during the pandemic remains pending. The main protectors of professional quality of life included training in several skills, such as specific training in palliative care, coping with death, stress management, self-care, and self-compassion (Galiana et al., 2022; Holland & Neimeyer, 2005; Sansó et al., 2018). Research suggests that specific training on death and dying increases professionals’ easiness with death-related issues and the care of dying patients (Holland & Neimeyer, 2005). Along the same lines, being versed in coping with death has been linked to higher levels of compassion satisfaction and lower levels of burnout and compassion fatigue in many studies (i.e., Galiana et al., 2022). Recent research also suggests that interventions aimed at improving stress management are relevant tools for preventing burnout and compassion fatigue and, consequently, for improving professional quality of life (Sansó et al., 2018). Indeed, professionals who question their abilities may feel threatened and seek to avoid activities connected to this domain, which in terms of end-of-life care may lead to overwhelmed professionals and a lack of professional care. The practice of self-care has also been found to be important in coping with work-related stressors among healthcare professionals in general (Sorenson et al., 2016) and among palliative care professionals in particular (Galiana et al., 2022). Self-compassion has been linked to a more adaptive psychological profile, and self-compassionate healthcare professionals can bolster resilience against stress and burnout (Raab, 2014). As a consequence, self-compassion has been associated with professional quality of life (Galiana et al., 2022).

Studying the effects of such a plethora of variables is crucial to understand how the therapeutic relationship has been affected in the presence of suffering during the COVID-19 pandemic. Additionally, professional quality of life has recently been linked to healthcare professionals’ wellbeing (Galiana et al., 2022) and to enhanced quality in terms of care (Salyers et al., 2017). Therefore, understanding the quality of life of professionals, including existing profiles and their behaviour with regard to sociodemographic, occupational, and inner life variables, can help us to understand and improve both the wellbeing of palliative care professionals, as well as the quality of care they provide.

### Aim of the study

In this context, this study’s purpose is twofold: firstly, to describe the professional quality of life profiles of a sample of Spanish palliative care professionals during the COVID-19 pandemic, including work satisfaction, burnout, compassion fatigue, and compassion satisfaction; and, secondly, to study the relationship between these profiles and sociodemographic variables, clinical situations experienced during the COVID-19 pandemic, protectors of professional quality of life, the quality of care provided by professionals, along with professionals’ wellbeing.

## Methods

### Study design

A cross-sectional survey of Spanish palliative care professionals was conducted to assess professional quality of life and several related variables.

### Setting and participants

The survey was conducted between March 2021 and April 2021. Professionals were accessed through the Spanish Society of Palliative Care (SECPAL). Participants were sampled from the SECPAL list of members, who were invited to complete an online survey using SurveyMonkey, a secure, anonymous online platform that also restricted multiple survey responses. Participation was voluntary and required informed consent from respondents.

A total of 338 palliative care professionals registered in the Spanish Directory of Palliative Care Resources (Sociedad Española de Cuidados Paliativos [SECPAL], 2016) were contacted by email, with two reminders sent over three-week intervals. In all invitations, professionals were encouraged to share and distribute the survey among their colleagues. The necessary sample size was not estimated a priori. In turn, a traditional rule of thumb in the context of structural equation modelling, which includes a minimum sample size of 200, was used (Kline, 2015).

For inclusion, the participants had to be healthcare professionals (physicians, nurses, psychologists, nursing assistants, social workers, or other) currently providing end-of-life care for patients, albeit not necessarily in palliative care settings.

A total of 278 professionals responded to the survey, yielding a response rate of 82.24%. However, as we asked professionals to distribute the survey, this figure is merely an approximation. After discarding participants that did not meet the inclusion criteria and those with missing values on the main outcomes (professional quality of life), 241 participants remained. The mean age was 45.34 years old (SD = 10.92); 77.2% were women. In terms of professional background, 47.7% were nurses, 38.2% were physicians, 5.4% were nursing assistants, 5.4% were psychologists, 1.2% were social workers, and 2.1% had other occupations. The majority of participants were married or living with their partners (67.6%). Table 1 compiles the sample characteristics.

**Table 1:** Sample characteristics

Variables	Categories	N	%
Gender	Men	55	22.8
	Women	186	77.2
Marital status	Single	50	20.7
	Married/living with a couple	163	67.6
	Divorced	26	10.8
	Widowed	2	0.8
Discipline	Nurse	115	47.7
	Physician	92	38.2
	Nursing assistant	13	5.4
	Psychologist	13	5.4
	Social worker	3	1.2
	Others	5	2.1

### Measures

Professional quality of life was measured using the following scales:

- The validated Spanish version of the Professional Quality of Life Scale (Short-ProQOL) (Galiana et al., 2020). The ProQOL comprises three subscales: compassion satisfaction, compassion fatigue, and burnout (Stamm, 2010). Each dimension is represented in the scale by three items that are scored using a 5-point Likert scale (ranging from 1 ‘never’ to 5 ‘very often’). The scores for each dimension are calculated as the sum of the three items and therefore range from 3 to 15. Reliability estimates in this study were 0.831 for compassion satisfaction, 0.789 for compassion fatigue, and 0.848 for burnout.
- The Maslach Burnout Inventory—Human Services Survey (MBI) (Maslach & Jackson, 1986). This is a 22-item questionnaire containing three constructs of burnout: emotional exhaustion (9 items), depersonalisation (5 items), and personal accomplishment (8 items). Each item is rated on a seven-point Likert scale depending on how frequently respondents experience a given feeling: from 0 (‘never’) to 6 (‘every day’). Reliability estimates for the sample were 0.919 for emotional exhaustion, 0.797 for depersonalization, and 0.826 personal accomplishment.
- The General Work Satisfaction Scale from the Michigan Organisational Assessment Scale (Cammann et al., 1983). The scale consists of three items. Each item is scored on a five-point Likert scale ranging from 1 (completely disagree) to 5 (completely agree). The estimated reliability was 0.829.
  We also gathered information on the following variables:
- Several sociodemographic characteristics, including age, gender, discipline, and marital status.
- COVID-19-related experiences, including the lack of access to suitable protective equipment, changes to workload and teamwork as a result of the pandemic, treatment of COVID-19 patients, deceased patients, family care, and professional end-of-life care for COVID-19 patients, and the right to a dignified death. The indicators used were: a) “*Have you been supplied with the necessary material resources (masks, gloves, PPE, *etc*.) since the onset of the pandemic?*”, measured using a YES/NO response option; b) changes in workload: “*How would you describe your workload since the onset of the pandemic?*”, measured using a five-point Likert scale ranging from 1 (‘it has decreased considerably’) to 5 (‘it has increased considerably’); c) changes in teamwork: “*What has teamwork been like (coordination between the different members of the interdisciplinary team, participation in decision-making, *etc*.) since the onset of the pandemic?*”, measured using a five-point Likert scale ranging from 1 (‘it has worsened considerably’) to 5 (‘it has improved considerably’); d) “*Have you treated COVID-19 patients in your unit or service?*”, measured using a YES/NO response option; e) “*Have any of these individuals died?*”, measured using a YES/NO response option—this indicator was answered only by professionals who had previously answered affirmatively to the question “*Have you treated COVID-19 patients in your unit or service?*”—; f) “*In general, has the dying process of COVID-19 patients been eased by the presence of a family member?*”, with a YES/NO response option; g) “*In general, do you think you have been able to provide adequate support to your COVID-19 patients in the final stages of their lives?*”, with a YES/NO response option; and h) “*In general, do you think that COVID-19 patients have had a dignified death?*”, with a YES/NO response option—indicators f), g), and h) were answered only by professionals who had previously answered affirmatively to the question “*Have any of these people died?*”—.
- Training received in palliative care, coping with death and stress, or emotional management. The questions associated with these indicators were: “*Have you been trained in palliative care?*”, “*Have you been trained in coping with suffering and death?*”, “*Have you been trained in stress and emotional management?*”, each of them with a YES/NO response option.
- Self-care. This variable was measured using the Professional Self-Care Scale (PSCS; Galiana et al., 2015), which contains nine items assessing three dimensions of professionals’ self-care: physical, inner, and social. This scale was originally developed and validated in Spanish. Items are scored on a 5-point Likert scale ranging from 1 (totally disagree) to 5 (totally agree). The reliability of the dimensions in this sample were 0.831, 0.908, and 0.746, respectively.
- Coping with death. The Spanish short version of the Coping with Death Scale (CDS-S; Galiana et al., 2019) was used to measure professionals’ abilities in dealing with death and their knowledge concerning preparedness for death. This comprises 9 items scored on a 5-point Likert scale ranging from 1 ‘totally disagree’ to 5 ‘totally agree’. The reliability in this sample was 0.910.
- Self-compassion. The Spanish version of the Self-Compassion Scale – Short Form (SCS – SF; García-Campayo et al., 2014) was used. The SCS contains 12 items assessing three main components of self-compassion and their opposites: self-kindness/self-judgment, common humanity/isolation, and mindfulness/over-identification. Items are scored on a 5-point Likert scale ranging from 1 ‘almost never’ to 5 ‘almost always’. Through these dimensions, two general factors of overall self-compassion can be measured: positive and negative self-compassion. Reliability estimates in this sample were 0.806 and 0.873, respectively.
- Wellbeing. The Spanish version of the Personal Wellbeing Index (Pérez-Belmonte et al., 2021) was used. This scale measures personal wellbeing through eight items using a 5-point Likert scale ranging from 1 (very dissatisfied) to 5 (very satisfied). The scale showed adequate psychometric properties, with a reliability estimate of 0.908.
- Quality of care. Quality of care was assessed using two indicators: “*How would you rate the quality of care in your unit?*”, which uses a 4-point Likert scale ranging from 1 (‘poor’) to 4 (‘excellent’), and the degree of agreement with the sentence “*I am confident that my institution’s management will solve any issues that may arise in the care of my patients*”, measured using a Likert scale ranging from 1 (‘totally disagree’) to 4 (‘totally agree’). These indicators were adapted from the ones used in 12 European countries and the United States for the same purposes (Aiken et al., 2012).

### Data analysis

Descriptive statistics of the study variables were calculated using SPSS 26 software (IBM Corp, 2019). Subsequently, Mplus 8.7 (Muthén & Muthén, 2021) was used for latent mixture modelling (LMM), which facilitates finding classes or profiles according to a number of variables, in this case variables related to professional quality of life. Specifically, several latent profile analyses (LPAs) were estimated. An LPA is a sophisticated variant of cluster analysis (Nylund et al., 2007) used to examine similarities and differences between individuals in relation to a number of quantitative variables. LPAs can be used in a confirmatory way when there are empirical and/or theoretical reasons behind a certain number of profiles, or in an exploratory fashion, when researchers seek to find profiles without any prior information. In this case, LPAs were conducted in an exploratory manner, and subgroup membership were inferred from the data without prior information on the optimal number of groups (Muthén, 2007). When used exploratorily, the rules determining the optimal number of classes must be explicitly stated. In this study, the number of classes kept were based on both statistical criteria and theoretical interpretability. The statistical criteria included information criteria, entropy, and statistical tests. The information criteria were the Bayesian Information Criteria (BIC), the sample size—adjusted BIC (ABIC), and the Akaike Information Criterion (AIC), with smaller values indicating a better fit. Entropy is an index assessing accuracy, ranging from 0 to 1 (perfect accuracy), with values above 0.7 considered reasonable and values above 0.8 deemed good. Additionally, the Lo–Mendell–Rubin (LMR) statistical test (Lo et al., 2001) was used to compare the improvement between neighbouring class models (i.e., two vs three classes, three vs four classes, and so forth). Statistically significant results indicate that the extra class improves appropriacy. In addition to statistical criteria, the selection of the number of classes remains subjective and requires theoretical and/or practical justifications, making the interpretability of the results of utmost importance (Lukočienė et al., 2010). LPA models were explored using the robust (full information) maximum likelihood estimation (MLR) method, an estimation procedure for handling missing data using all available data points (Little et al., 2014).

Once the number of profiles was determined using LPAs, participants were included in their most likely profile and these profiles were then compared on a number of variables of interest using chi-squared and *t*-tests for independent samples through SPSS 26 (IBM Corp, 2019).

## Results

### Latent profile analyses

Three scales were used to study the professional quality of life profiles: the Short-ProQOL, the MBI, and the General Work Satisfaction Scale. These scales included seven subscales or dimensions measuring the positive pole of professional quality of life (compassion satisfaction, personal accomplishment, and work satisfaction) and the negative pole (compassion fatigue, burnout, emotional exhaustion, and depersonalisation). Table 2 shows the descriptive statistics for these variables.

**Table 2:** Descriptive statistics for the variables measuring professional quality of life

Variable	M	SD	Min	Max
Compassion satisfaction	13.86	1.44	7.00	15.00
Burnout	8.22	2.36	3.00	15.00
Compassion fatigue	6.97	2.08	3.00	15.00
Emotional Exhaustion	18.17	9.07	2.00	48.00
Depersonalization	4.82	3.63	0.00	17.00
Personal Accomplishment	33.82	6.19	0.00	47.00
Work satisfaction	4.42	0.62	1.67	5.00

*M* Mean, *SD* Standard deviation, *Min* Minimum score, *Max* Maximum score

Five LPAs were estimated, from one profile (or baseline) to five latent profiles or classes. The best-fit solution was the one with the lowest information criteria (BIC, ABIC, and AIC), the highest entropy value, and a statistically significant LMR. The interpretability of the results was also taken into consideration. However, the criteria were somewhat contradictory. Two, three, and five profiles obtained good entropy values. The information criteria favoured the more complex solution (see Table 3). However, the LMR test found no gain through including more than two classes. Considering that entropy was adequate for the model with two profiles and that it was highly interpretable, this solution was maintained.

**Table 3:** Models fit for one, two, three, four, and five latent profiles

Model	AIC	BIC	ABIC	Entropy	LMR	p
One-profile model	7723.443	7772.231	7727.853	NA	NA	NA
Two-profiles model	7375.409	7452.074	7382.339	.818	355.923	0.001
Three-profiles model	7270.611	7375.155	7280.061	.833	118.106	0.153
Four-profiles model	7214.298	7346.720	7226.268	.788	70.702	0.236
Five-profiles model	7163.332	7323.632	7177.822	.820	65.474	0.305

*AIC* Akaike Information Criterion, *BIC* Bayesian Information Criterion, *ABIC* Adjusted BIC, *LMR* Lo–Mendell–Rubin test, *NA* Not applicable

A first step after the creation of the classes was to test whether they showed statistically significant differences on all variables used in the LPA. Therefore, we tested the mean differences between the two classes firstly on all variables with a MANOVA, which was statistically significant (*F*(7, 212) = 59.700, *p* < 0.001), and then with follow-up *t*-tests for the individual variables. All *t*-tests were statistically significant, and their effect sizes were large, suggesting that all variables help in distinguishing between the two classes. Table 4 presents these *t*-tests and Cohen’s effect size measures.

**Table 4:** Means and t tests for the variables in each latent profile

	Low professional quality of life	High professional quality of life	*t*	*p*	Cohens’ *d*
*N* (%)	*N* = 79; 32.78%	*N* = 162; 67.22%			
Compassion satisfaction	12.96	14.36	-7.92	< .05	1.09
Burnout	10.31	7.10	13.68	< .05	1.78
Compassion fatigue	8.69	6.03	12.54	< .05	1.51
Emotional exhaustion	26.56	13.57	14.64	< .05	2.07
Depersonalization	7.32	3.45	8.91	< .05	1.26
Personal accomplishment	30.27	35.76	-6.77	< .05	0.96
Job satisfaction	4.00	4.64	-8.42	< .05	1.19

As for the two resulting profiles, Profile 1 included 32.78% of the sample and Profile 2 included the remaining 67.22%. Table 4 shows the means for the variables of both profiles. The first profile is characterised by lower means on the “positive” markers of professional quality of life (compassion satisfaction, personal accomplishment, and work satisfaction) and higher means on the “negative” markers (burnout, compassion fatigue, emotional exhaustion, and depersonalisation). Consequently, this profile was referred to as *low professional quality of life*. Conversely, the second profile, which had higher means in the positive pole of professional quality of life and lower means in the negative dimensions of professional quality of life, was referred to as *high professional quality of life*. Considering the effect sizes, the differences between the two classes were more salient in emotional exhaustion and burnout.

### Relationships between professional quality of life profiles and sociodemographic variables

Once the latent profiles were established and the professionals were classified into their more likely profile, the professional quality of life profiles were linked to the sociodemographic characteristics of the participants. These sociodemographic variables included sex, age, discipline, and marital status.

Age differences between the two professional quality of life profiles were explored using a *t*-test for independent samples. The results unearthed statistically significant differences (*t*(234) = -3.275; *p* < 0.001), with participants in the high professional quality of life group being older (*M* = 46.93; SD = 10.31) compared to participants in the low professional quality of life group (*M* = 42.06; SD = 11.48).

The relationship between sex and the quality of life profiles was examined using a chi-squared test, which yielded no statistically significant differences: *χ*^*2*^(1) = 2.704; *p* = 0.100; Cramer’s *V* = 0.106. Similarly, no relationship was identified between the professional quality of life profiles and marital status: *χ*^*2*^(2) = 0.769; *p* = 0.681; Cramer’s *V* = 0.057. The same strategy was used to study the relationship between professional quality of life profiles and discipline (clinicians vs nurses), with statistically significant results: *χ*^*2*^(1) = 5.996; *p* = 0.014; Cramer’s *V* = 0.170. Pearson’s residuals indicated a higher likelihood of a low professional quality of life profile for nurses, while clinicians were more likely to reach a high professional quality of life profile.

### Impact of clinical situations experienced during the COVID-19 pandemic on professional quality of life

In order to study the relationship between professional quality of life and pandemic-derived clinical situations, two *t*-tests were performed. The first test showed no statistically significant differences in workload means during the pandemic between the low and high professional quality of life profiles (*t*(239) = 1.081; *p* = 0.281). However, the second test identified statistically significant differences in teamwork (*t*(239) = -2.347; *p* = 0.020). That is, while professional quality of life, in terms of profile, was not linked to changes in the level of workload caused by the pandemic, such changes did occur in teamwork. Specifically, professionals with a high professional quality of life underwent no change in their teamwork (*M* = 2.99; SD = 1.14), while those with a low professional quality of life reported a decrease in their teamwork insomuch as this affects coordination and decision-making (*M* = 2.60; SD = 1.26).

No statistically significant relationships were identified between professional quality of life and the following experiences, with the exception of dignified death: lack of access to adequate protective equipment (*χ*^*2*^(1) = 2.157; *p* = 0.142; Cramer’s *V* = 0.095), treatment of COVID-19 patients (*χ*^*2*^(1) = 0.306; *p* = 0.580; Cramer’s V = 0.036), deceased patients (*χ*^*2*^(1) = 0.931; *p* = 0.335; Cramer’s *V* = 0.064), family care (*χ*^*2*^(1) = 2.912; *p* = 0.088; Cramer’s *V* = 0.116), and professional end-of-life care for COVID-19 patients (*χ*^*2*^(1) = 3.032; *p* = 0.082; Cramer’s *V* = 0.118). Professionals who felt that their patients had been allowed a dignified death were more likely to have a high quality of life profile compared to those who felt that patients were unable to have a dignified death (*χ*^*2*^(1) = 7.665; *p* = 0.006; Cramer’s *V* = 0.186). It is also important to note that participants who reported that their patients were accompanied by their family members, or a healthcare professional were also more likely to have a high professional quality of life profile, with these associations being marginally significant.

### Relationships between protectors of professional quality of life and professional quality of life

In order to study the relationship between specific training and professional quality of life profiles, three chi-squared tests were performed, one for palliative care training (*χ*^*2*^(1) = 4.996; *p* = 0.026; Cramer’s *V* = 0.144), one for training in coping with death (*χ*^*2*^(1) = 10.751; *p* = 0.001; Cramer’s *V* = 0.211), and one for training in stress and emotions (*χ*^*2*^(1) = 10.230; *p* = 0.001; Cramer’s *V* = 0.206), all showing statistically significant associations. In all cases, higher professional quality of life was related to specific training, with more pronounced associations between professional quality of life and training in coping with death and training in stress and emotions.

Several *t*-tests were performed to determine the relationship between professional quality of life and the ability to cope with death, self-care, and self-compassion. In all cases the results were statistically significant (see Table 5). Participants with a high professional quality of life profile were greater skilled in coping with death and attained higher levels of self-care and positive self-compassion, and lower levels of negative self-compassion.

**Table 5:** Means and t tests for coping with death, self-care, self-compassion, and well-being in each latent profile

	Low professional quality of life	High professional quality of life	*t*	*df*	*p*
Coping with death	3.71	4.09	-4.37	216	< .001
Physical self-care	3.19	3.77	-3.98	217	< .001
Psychological self-care	2.68	3.12	-2.670	217	.008
Social self-care	3.64	4.29	-6.497	217	< .001
Positive self-compassion	2.99	3.50	-5.508	232	< .001
Negative self-compassion	3.41	2.56	7.693	232	< .001

### Relationships between professional quality of life profiles and professionals’ wellbeing and quality of care

The relationship between professional quality of life profiles and professionals’ wellbeing was studied using a *t*-test, which identified a statistically significant association between these variables: *t*(213) = -4.127; *p* < 0.001. Participants with a high professional quality of life (class 2) showed higher levels of wellbeing (*M* = 4.05; SD = 0.51) compared to those with a low professional quality of life (*M* = 3.73; SD = 0.59).

Finally, two chi-squared tests were performed to explore the association between professional quality of life and quality of care and trust in the institution. In both cases, results indicated that professionals with a low professional quality of life profile were more likely to rate the quality of care of their unit as poor compared to those with a high professional quality of life (*χ*^*2*^(3) = 14.551; *p* = 0.002; Cramer’s *V* = 0.260), who also trusted their institution’s ability to solve patient care problems less (*χ*^*2*^(3) = 14.703; *p* = 0.002; Cramer’s *V* = 0.262).

## Discussion

This study intended to gain a deeper understanding of the professional quality of life of palliative care professionals through the description of professional quality of life profiles, alongside the study into the relationship between these profiles and sociodemographic variables, clinical situations experienced during the COVID-19 pandemic, the protectors of professional quality of life, and professionals’ quality of care and wellbeing.

To this end, we have studied the quality of life profiles of professionals using latent profile analysis. As far as can be ascertained, this is the first study to examine palliative care professionals' quality of life in such a comprehensive way. A number of studies have also used latent profile analysis to investigate factors influencing the well-being of healthcare professionals, yet none of these have sought out the broader concept of professional quality of life. For example, Eley et al. (2016) studied personality traits in 808 Australian medical students, and Park et al. (2021) assessed emotional labour, burnout and turnover intentions in a sample of 204 nurses working in university hospitals in South Korea, both studies using latent profile analysis.

Our results suggested the existence of two professional quality of life profiles. The most predominant, Profile 2 or *high professional quality of life*, was identified in two-thirds of the sample. It was characterised by high levels of compassion satisfaction and personal accomplishment, and low levels of compassion fatigue and burnout. The other profile, Profile 1 or *low professional quality of life*, was characterised by medium levels of burnout and high levels compassion fatigue; levels of compassion satisfaction and work satisfaction were lower than the ones obtained in Profile 2, while still high. This is in line with previous studies performed with Spanish palliative care professionals, which have reported an adequate professional quality of life for most of them (Galiana et al., 2017). Compared to other health areas, where COVID-19 has increased the prevalence of burnout, ranging from 30 to 60% (Barello et al., 2020), and compassion fatigue (Blanco-Donoso et al., 2021; Ruiz-Fernández et al., 2020; Samaniego et al., 2020), the professional quality of life of palliative care professionals has not been affected as much.

With regard to the second study aim, the results revealed the main factors influencing professionals’ quality of life during the COVID-19 pandemic. On the one hand, the results of the sociodemographic variables study failed to identify differences by sex or marital status, as in previous studies, in which none of these variables were related to professional quality of life (i.e., Ruiz-Fernández et al., 2020). On the other hand, other authors did find an association between professional quality of life and sex. Specifically, in a study by Samaniego et al. (2020), being female was a factor that negatively influenced professional quality of life, with women undergoing greater compassion fatigue than men.

In terms of sociodemographic variables, a relationship was found between age and professional quality of life. Specifically, professionals with a high quality of life profile were older than those with a low quality of life profile. This concurs with a study by Azoulay et al. (2020), in which younger professionals were those with the highest burnout rates. However, just the opposite was found in another study in which older professionals had the lowest professional quality of life scores (Dosil et al., 2020). In terms of professions, in light of our results, physicians were more likely to enjoy a better professional quality of life than nurses. The discipline effect has been noted in previous studies (Chor et al., 2021), but there is still some controversy. In fact, two recent studies found that physicians obtained the poorest results in terms of professional quality of life (Dosil et al., 2020; Ruiz-Fernández et al., 2020).

The study also analysed the bearing of clinical situations experienced during the pandemic, such as changes to workload, difficulties in accessing PPE, caring for COVID-19 patients, changes in teams, and the inability to support the patient at the time of death. On the one hand, no association was found between professional quality of life profiles and the experience of changes to their workload, nor with hindrances in accessing protective equipment. These results are somewhat counter-intuitive and deviate from previous studies, in which lack of PPE (Moreno-Mulet et al., 2021) and increased workload (Blanco-Donoso et al., 2021) were associated with professional quality of life. It is also important to note that the samples in these studies were made up of front-line healthcare workers rather than palliative care professionals. Furthermore, in the study sample, no relationship was identified between caring for patients with COVID-19, despite their eventual death, and professional quality of life. When surveying the impact of the pandemic on healthcare professionals in previous studies, evidence suggests that caring directly for patients with COVID-19 constitutes a risk factor for the professional’s quality of life, as well as for distress-related conditions, namely anxiety and depression (Ruiz-Fernández et al., 2020). This difference may be due to the fact that these studies were not specifically conducted with palliative care professionals, moreover with hospital and primary care professionals, who are less accustomed to working with patients at the end of life stage. For palliative care professionals, COVID-19 patients did not pose as great a challenge as for other healthcare workers, as this professional profile is well versed at working with suffering and uncertain outcomes.

Conversely, the clinical situations that have been shown to influence professional quality of life are teamwork and the healthcare professional’s perception that the patient has died surrounded by loved ones or by a professional and has died a dignified death. These results are partially consistent with those of a study by Moreno-Mulet et al. (2021), conducted with ICU professionals, where a statistically positive relationship was identified between higher levels of compassion satisfaction and having been able to provide adequate support and follow-up to the patient at the time of death, but not between the negative dimensions of professional quality of life (burnout and compassion fatigue). According to Mitchinson et al. (2021) findings, current results outline in which extent professional quality of life is deteriorated due to the adverse combination of job preassure and reduced human interaction.

Other notable results relate to protectors of professional quality of life. In the study, professionals who were more likely to enjoy a high professional quality of life profile were those with higher education in all the surveyed areas, yet especially in training in the ability to cope with death and in managing stress and emotions. These results are in line with previous studies (Sansó et al., 2018), in which training in emotional balance, meditation, and other contemplative practices resulted in increased professional quality of life. Other interventions focused on stress-reduction, such as the one developed by Bruneau and Ellison (2004), have also shown a positive impact on professional quality of life, particularly burnout decrease. In the case of the ability to cope with death, our results are in line with the ones presented by Melo and Oliver (2010), who found that a course to reduce death anxiety improved professional quality of life.

Furthermore, obtaining high scores in ability to cope with death, practising self-care (in all dimensions: inner, social, and physical), and having high levels of positive self-compassion and low levels of negative self-compassion are common factors among professionals with a high professional quality of life profile. These variables match those that have been displayed as protectors of professional quality of life in several previous studies (Galiana et al., 2017, 2022; Sansó et al., 2020). For example, Horn and Johnston (2020) have recently gathered a bulk of academic evidence corroborating the relationship between engaging in self-care activities, such as exercise, mindfulness, adequate time of sleep, or adequate time off, and higher levels of professional quality of life. In this same line, previous evidence on the relation between ability to cope with death and professional quality of life is abundant and comes from different countries and continents (Oliver et al., 2021). When it comes to self-compassion, evidence is also large and quite recent. Indeed, the review carried out by Garcia et al. (2021) found that self-compassion has been associated with increased capacity for self-care, mindfulness, and professional quality of life, and a decrease in perceived burnout risk and secondary traumatic stress. Evidence of the impact of these variables on professional quality of life is particularly important, given that most healthcare professionals are unaware of them and have not been trained in this regard, and results on said relationship during the COVID-19 pandemic remain scant.

Additionally, professional quality of life and wellbeing appear to be closely linked, as professionals who have a higher level of professional quality of life also have high levels of wellbeing. This close-knit relationship has also been observed in previous studies (Galiana et al., 2022). For example, Kase et al. (2019) found that compassion fatigue and burnout directly influenced the well-being and professional performance of palliative care providers. Sansó et al. (2020) also found a close relationship between professional quality of life and wellbeing, with the first explaining almost 60% of the variance of the latest. Indeed, a detriment of the professional quality of life of healthcare personnel can have important consequences on their personal wellbeing (Koh et al., 2015; Sansó et al., 2020). In order to avoid such detriment, it is imperative for the development of optimal states of wellbeing that health professionals have work and personal resources to turn work demands into a source of learning and professional and personal growth that gives meaning to the work done (Donoso et al., 2015; Smith et al., 2015).

Finally, professionals with a more enhanced profile of quality of life also perceived that they provide a higher quality of care in their service while placing greater trust in their institutions to address issues that may arise during their patients’ care. Although research on this matter is scarce in the palliative care context, evidence on the relation between professional quality of life and quality of care has been deeply studied in other health areas. This relation has been established in terms of best practices, communication, medical errors, patient outcomes, and quality and safety, specifically in relation to burnout (Tawfik et al., 2019). The results found in this study, in line with the one just pointed, evidence that professional quality of life is not only necessary for professionals’ wellbeing, but also for care excellence.

Although this study has several strengths, such as the originality of its approach, the use of a sophisticated methodology, and the development of a very comprehensive approach to professional quality of life, it also features a number of drawbacks. Firstly, the sample was incidental and, as a consequence, the representativeness of the results may be compromised. Secondly, variables that may influence professionals’ quality of life, such as clinical experience or time spent caring for dying patients were not included in the survey. Finally, another shortcoming is the cross-sectional nature of the study. The difficulties in establishing paths in data gathered at a single point in time are well known, though also difficult to overcome. Future studies with a longitudinal design are needed to appraise the causal links between these complex aspects of professional quality of life.

## Conclusions

In conclusion, our results identified two profiles describing the professional quality of life of palliative care workers: a high profile, with adequate levels, which was present in the majority of study participants; and a low profile, in which burnout levels were medium, and compassion fatigue levels were high, representing up to 30% of the sample. Although this group did not display extreme quality of life problems, our results should be taken seriously. As recently stated by the WHO (2022), “in the absence of targeted policy action, there is a risk that the pressures of COVID-19 will exacerbate long-standing shortcomings related to healthcare shortages and difficulties in attracting and retaining healthcare workers” (p. viii).

Today, the healthcare system needs more than ever to take care of its professionals owing to the implications that their wellbeing and quality of life have on the quality of care they provide. This study unveils the profile of professionals who have a better professional quality of life so that lessons can be learnt from their experiences. Providing professionals with education and training to improve their ability to cope in the face of death, dealing with stress, maintaining cohesive teams, and working for organisations that build trust among their professionals are some of the keys to maintaining an optimal level of wellbeing among healthcare professionals. Caring for carers is bottom-line for maintaining healthy healthcare systems.

## Data Availability

The data that support the findings of this study are available from the corresponding author upon reasonable request.
